# Enhancing the Elevated-Temperature Mechanical Properties of Levitation Melted NbMoTaW Refractory High-Entropy Alloys via Si Addition

**DOI:** 10.3390/ma18153465

**Published:** 2025-07-24

**Authors:** Yunzi Liu, Xiaoxiao Li, Shuaidan Lu, Jialiang Zhou, Shangkun Wu, Shengfeng Lin, Long Wang

**Affiliations:** 1 School of Materials and Chemical Engineering, Xi’an Technological University, Xi’an 710021, China; liuyunzi@xatu.edu.cn (Y.L.); lixiaoxiao@foxmail.com (X.L.); zjl010914@163.com (J.Z.); wushangkun_xatu@163.com (S.W.); linshengfeng@foxmail.com (S.L.); 2 Zhijian Laboratory, Rocket Force University of Engineering, Xi’an 710025, China

**Keywords:** refractory high-entropy alloys, induction levitation melting, microstructural characterization, mechanical properties, wear resistance

## Abstract

To enhance the mechanical properties of NbMoTaW refractory high-entropy alloys (RHEAs), Si was added at varying concentrations (*x* = 0, 0.25, and 0.5) via vacuum induction levitation melting (re-melted six times for homogeneity). The microstructure and mechanical properties of NbMoTaWSi*_x_* (*x* = 0, 0.25, and 0.5) RHEAs were characterized using scanning electron microscopy (SEM), universal testing, microhardness testing, and tribological equipment. Experimental results manifested that Si addition induces the formation of the (Nb,Ta)_5_Si_3_ phase, and the volume fraction of the silicide phase increases with higher Si content, which significantly improves the alloy’s strength and hardness but deteriorates its plasticity. Enhanced wear resistance with Si addition is attributed to improved hardness and oxidation resistance. Tribological tests confirm that Si_3_N_4_ counterfaces are optimal for evaluating RHEA wear mechanisms. This work can provide guidance for the fabrication of RHEAs with excellent performance.

## 1. Introduction

The accelerated advancement of aerospace technologies has driven extensive research on Ni-based superalloys, prized for their exceptional oxidation resistance and corrosion stability under extreme thermal conditions [1,2,3]. However, these conventional superalloys face fundamental limitations in meeting the escalating operational demands of modern propulsion systems, particularly under a combination of extreme environments featuring temperatures exceeding 1000 °C, hypersonic velocities, and extreme mechanical loading [4,5,6,7]. This performance gap underscores the critical need for developing next-generation high-temperature materials capable of enabling next-generation aircraft engine technologies. In order to address this issue, a novel alloy, the refractory high-entropy alloy (RHEA), was proposed by Senkov [8] to provide a vast design space and enable precise control strategies for tailoring properties in advanced alloys. RHEAs generally have single body-centered cubic (BCC) structures, high melting points, and strength at elevated temperatures, combining exceptional thermal stability with remarkable strength retention at temperatures approaching 80% of their melting points [9,10,11,12,13,14]. The intrinsic high melting temperatures (>2000 °C) of RHEAs directly translate to enhanced maximum service temperatures, positioning them as prime candidates for ultra-high-temperature structural applications [15,16,17,18]. Among notable RHEA systems, the NbMoTaW and VNbMoTaW alloys demonstrate exceptional high-temperature mechanical performance, maintaining compressive strengths exceeding 400 MPa at 1600 °C [19,20,21]. Compared to the significant yield strength reduction (>40%) observed in conventional high-temperature alloys from room temperature to 600 °C, RHEAs exhibit a much smaller strength degradation of only 20–40%, owing to their unique solid solution strengthening and sluggish diffusion effects [20,21,22]. It should be noted that RHEAs lack sufficient oxidation resistance at elevated temperatures [22,23,24], which limits their prospects for long-term use in practical applications. Consequently, significant efforts have been dedicated to identifying effective approaches to enhance the high-temperature oxidation performance of RHEAs [21,22,23,24,25]. Among various technological approaches, microalloying has emerged as an effective strategy to balance the comprehensive properties of alloys. Lu et al. [26] demonstrated the efficacy of Y microalloying in AlMo_0.5_NbTa_0.5_TiZr RHEAs, where Y-doping was shown to suppress refractory element volatilization during oxidation and improve oxide scale adherence. Si has garnered particular attention as a strategic alloying element, with Franz et al. [27] revealing that Si additions in Ta-Mo-Cr-Ti-Al systems promote the formation of protective (Cr,Al)-rich oxide layers at temperatures exceeding 1100 °C. Their findings revealed a positive correlation between Si content and oxidation resistance enhancement, particularly regarding mechanical stability under thermal exposure. Beyond oxidation resistance, Si plays a multifunctional role in RHEA microstructural engineering. It facilitates precipitation hardening through the controlled formation of intermetallic phases, including B2-type structures [28,29], Laves phases [30,31], and silicide compounds [32,33]. This phase engineering capability addresses the inherent limitations of BCC-phase strengthening in conventional RHEAs, which often results in compromised mechanical performance [33,34,35].

The development of Nb-Si-based alloys exemplifies this strategy, combining advantageous characteristics such as reduced density, enhanced low-temperature ductility, and superior high-temperature strength with improved oxidation resistance [36,37,38,39]. Crucially, the mechanical benefits of Nb-Si reinforcement phases become maximized when maintained within optimal volume fractions, highlighting the importance of precise composition control.

In this investigation, we employ vacuum induction levitation melting (VILM)—an advanced processing technique offering distinct advantages over conventional vacuum arc melting. The VILM method enables superior homogenization of high-melting-point alloys (>2000 °C) while facilitating rapid volatilization of high-vapor-pressure impurities, thereby enhancing ductility through purity control. Utilizing this technique, we fabricated NbMoTaWSix (*x* = 0, 0.25, and 0.5) RHEAs with homogenized microstructures and systematically evaluated their phase evolution, mechanical properties, and high-temperature performance (including oxidation and ablation resistance). This comprehensive study provides fundamental insights into the microalloying mechanisms of Si in RHEAs, while establishing a framework for designing next-generation ultrahigh-temperature alloys through strategic phase engineering.

## 2. Materials and Methods

The NbMoTaWSix (*x* = 0, 0.25, and 0.5) RHEAs were synthesized via vacuum induction levitation melting (VILM) using the raw materials of Nb, Mo, Ta, W, and Si with purities exceeding 99.9 wt.%. Each 900 g ingot underwent sixfold remelting to ensure its compositional homogeneity. Following melting, furnace cooling was employed to obtain cylindrical as-cast alloys measuring 50 mm in diameter and 35 mm in height. Specimens (5 mm × 5 mm × 10 mm) were extracted from the ingot core via wire electrical discharge machining for characterization and testing, followed by sequential polishing with 400# to 2000#-grit Si carbide abrasive paper and ultrasonic cleaning in anhydrous ethanol. Microstructural and crystallographic analyses were conducted using X-ray diffraction (XRD, Bruker (Billerica, MA, USA) D8 Advance, Cu-Kα radiation, λ = 1.5406 Å) and field-emission scanning electron microscopy (FE-SEM, Quanta FEG 450 equipped with EDS).

Uniaxial compression testing was conducted at room temperature using a computer-controlled universal testing machine (UTM5105X, SUNS, Shenzhen, China) on prismatic specimens with nominal dimensions of 6 mm × 6 mm × 10 mm, maintaining a constant strain rate of 1.0 × 10^−3^ s^−1^ at room temperature. To ensure the test results, each surface of the sample was sanded to 2000 grit with sandpaper. Five replicate tests per composition were performed to ensure statistical reliability, with mean values reported for the yield strength analysis. Vickers microhardness (HV_0_._5_) measurements were acquired using a digital microhardness tester (HV-1MD, Shanghai Hengyi, Shanghai, China) under standardized testing conditions: 500 gf load with a 15 s dwell time. Tribological characterization was performed on a ball-on-disk tribometer (HT-1000, Lanzhou Zhongke Kaihua Technology, Lanzhou, China) under dry sliding conditions. Specimens underwent ultrasonic cleaning in anhydrous ethanol and precision weighing (±0.1 mg) pre/post testing. Wear tests utilized GCr15 steel balls and Si_3_N_4_ balls as counterface materials under a load of 10 N, a rotational speed of 200 r/min, a friction radius of 2 mm, and a duration of 30 min at 25 ± 5 °C. In post-testing, wear scars were analyzed via SEM/EDS, and wear track morphology quantification was achieved through non-contact optical profilometry (ContourGT-X8, Bruker) with subsequent wear volume calculation.

## 3. Results and Discussion

### 3.1. Microstructure and Compositions of NbMoTaWSi_x_ RHEAs

**Figure 1:**
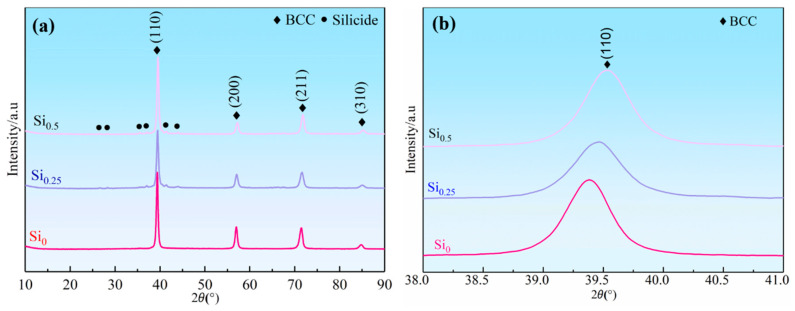
XRD patterns of (**a**) NbMoTaWSi*_x_* RHEAs with different Si content, (**b**) enlarged XRD patterns of NbMoTaWSi*_x_* RHEAs.

Figure 1a presents the X-ray diffraction (XRD) patterns of the NbMoTaWSix RHEAs. The base NbMoTaW alloy exhibits a characteristic single-phase body-centered cubic (BCC) structure (Space group: 1m–3m), as evidenced by the absence of secondary phase peaks. The introduction of Si leads to significant microstructural changes, manifested as the appearance of new diffraction peaks corresponding to the M_5_Si_3_-type silicide (JCPDS 34-0376 for Nb_5_Si_3_ and 31-1433 for Ta_5_Si_3_). Notably, the relatively low intensity of these silicide peaks compared to the primary BCC phase (integrated intensity ratio < 0.15) suggests limited phase fraction formation. A systematic shift in the (110) BCC diffraction peak positions toward higher angles is observed with increasing Si content (Figure 1b), indicating progressive lattice contraction. Quantitative analysis via Bragg’s law reveals precise lattice parameter reductions from 3.238 Å (*x* = 0) to 3.231 Å (*x* = 0.25) and 3.227 Å (*x* = 0.5) [40]. The obtained results were caused by a decrease of the larger radius elements, Nb and Ta, in the matrix phase (see Table 1), and the formation of an M_5_Si_3_-type silicide.

**Figure 2:**
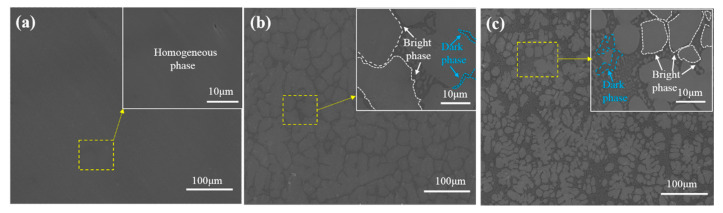
SEM images of the NbMoTaWSi*_x_* RHEAs with different Si content: (**a**) *x* = 0, (**b**) *x* = 0.25, and (**c**) *x* = 0.5.

The phase compositions of the NbMoTaWSix RHEAs were further analyzed by SEM/EDS, as shown in Figure 2a–c. The base NbMoTaW alloy exhibits a homogeneous single-phase microstructure with no discernible grain boundaries or secondary phases (Figure 2a), consistent with its BCC-dominated XRD pattern. Si introduction induces a pronounced phase segregation, manifested as discrete bright-phase precipitates (Figure 2b), identified via EDS as (Nb,Ta)-rich M_5_Si_3_ silicides. Quantitative image analysis reveals a Si-content-dependent refinement of these precipitates: average diameters decrease from 54.5 ± 3.2 μm (*x* = 0.25) to 21.6 ± 1.8 μm (*x* = 0.5), accompanied by a morphological transition from equiaxed to dendritic structures (Figure 2c).

**Figure 3:**
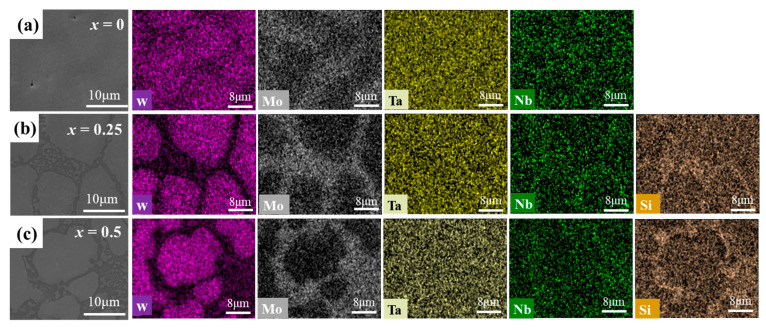
SEM micrographs and EDS mappings of the NbMoTaWSi*_x_* RHEAs with different Si content: (**a**) *x* = 0, (**b**) *x* = 0.25, and (**c**) *x* = 0.5.

The microstructure and EDS mapping of NbMoTaWSi*_x_* RHEAs are shown in Figure 3.. As can be seen in Figure 3a, Mo in the NbMoTaW RHEA is mainly segregated in the interdendritic region, with the W element occurring in a generally micro-degraded manner with a relatively low content between dendrites. Correspondingly, the distribution trends of Nb and Ta elements are not obvious. As presented in Figure 3b, as the Si content increases, the alloy exhibits a microstructure consisting of dendritic and interdendritic structures. It can be seen in Figure 3c that the dendrite regions mainly consist of W, and the interdendrite regions are mainly rich in Mo and Si elements. This can be attributed to the thermodynamics of the combination reaction, which always favors the formation of a stabilized structure to diminish the Gibbs free energy. The higher entropy favors the formation of solid solutions, and the lower enthalpy promotes the formation of intermetallic compounds. Although the above elements can participate in forming silicide, it is evident that the more negative mixing enthalpy between Nb, Ta, and Si favors the formation of intermetallic compounds [42,43,44]. Meanwhile, the mixing enthalpies of silicide between W, Mo, and Si are less competitive. This demonstrates that W and Mo atoms tend to be excluded from the silicide during the solidification process, resulting in a low distribution of Mo and W in the silicide. Hence, the silicide phase was finally identified as (Nb, Ta)_5_Si_3_.

### 3.2. Mechanical Properties of NbMoTaWSi_x_ RHEAs

**Figure 4:**
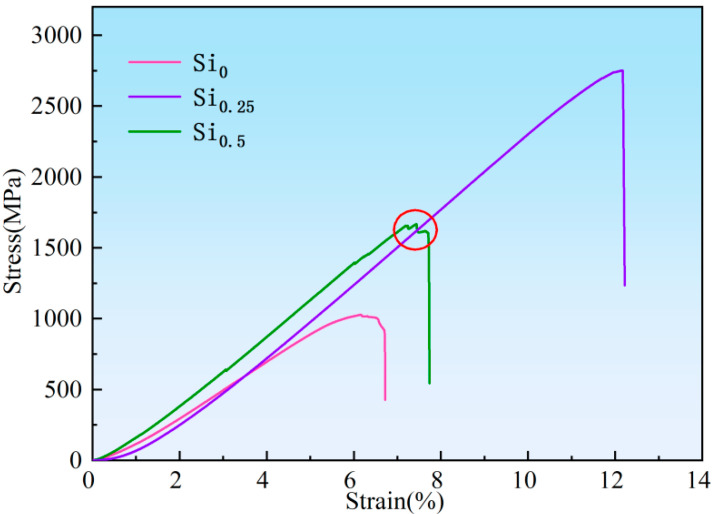
Compressive stress-strain curves of the NbMoTaWSi*_x_* RHEAs at room temperature.

The compressive engineering stress-strain curves of the NbMoTaWSi*_x_* RHEAs at room temperature are shown in Figure 4, while the typical mechanical properties of compression are listed in Table 2. It can be seen that the yield strengths of the NbMoTaW and NbMoTaWSi_0.25_ alloys exhibit 1029.36 MPa and 2560.45 MPa, respectively. This can be attributed to the fact that the addition of Si has a significant effect on improving the strength of RHEAs. However, when the Si content increases from 0.25 to 0.5, the yield strength decreases to 1560.83 MPa. This might be due to the increase in the volume of brittle silicates in NbMoTaWSi RHEAs, resulting in severe brittle fracture. Thus, the NbMoTaWSi_0.25_ RHEAs have the strongest yield strength in NbMoTaWSi*_x_* (*x* = 0, 0.25, and 0.5) RHEAs. Interestingly, compared with the specimens without Si, the bright phase and dendrite structure, light exposure, yield strength, and plastic strain of the NbMoTaWSi_0.25_ RHEA were significantly increased. Particularly, the plasticity of the NbMoTaWSi_0.25_ RHEA was increased by 87.7% compared with that without Si addition. Furthermore, the elastic modulus of the NbMoTaW alloy is 200.7 GPa, while the NbMoTaWSi_0.25_ and NbMoTaWSi_0.5_ alloys exhibit elastic moduli reaching 265.3 GPa and 249.4 GPa, respectively. Due to its high elastic modulus, the NbMoTaWSi_0.25_ RHEA can withstand elevated stress levels while undergoing less engineering strain at a given applied stress. Consequently, this RHEA exhibits relatively superior ductility.

**Figure 5:**
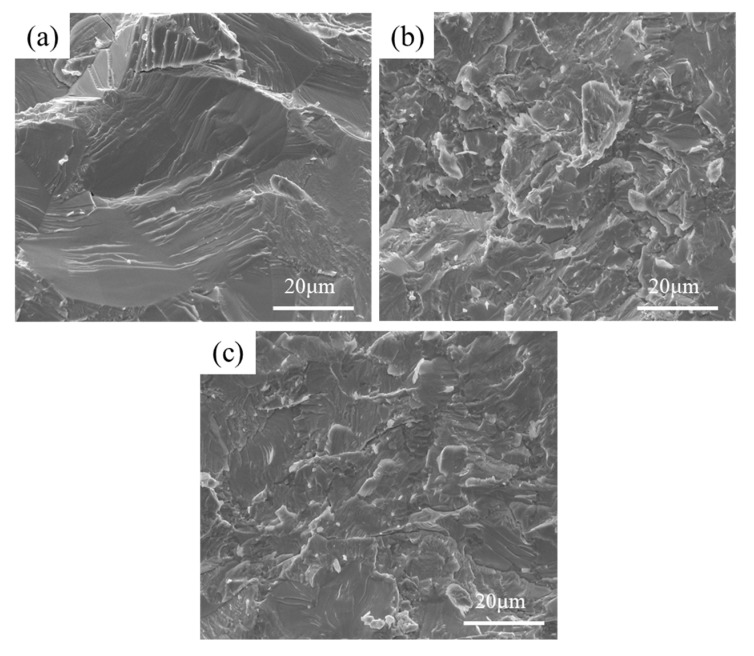
Fracture morphologies of the NbMoTaWSi*_x_* RHEAs with different Si content: (**a**) *x* = 0, (**b**) *x* = 0.25, and (**c**) *x* = 0.5.

Figure 5 presents the microstructures of the NbMoTaWSi*_x_* RHEAs after fracture at room temperature. Obviously, it exhibits river patterns on the fracture surface, which are characteristic of typical brittle cleavage fractures. The fracture behavior of the NbMoTaW RHEA is predominantly governed by the inherent brittleness of its BCC phase, as evidenced by the cleavage facets in Figure 5a. For NbMoTaWSi*_x_* RHEAs, the precipitated M_5_Si_3_-type phases will aggravate the brittle fracture of the refractory high-entropy alloy. This demonstrates that the coordinated action of the two fracture behaviors, evolving from the matrix and silicide, contributes to the fracture. It should be known that the silicide has a strong constraint effect on the matrix. When the volume fraction of silicide is relatively greater, it causes local inconsistencies in the fracture of RHEAs, resulting in a reduced plasticity of the alloy. As the content of Si increases, the fracture area increases dramatically. Therefore, the Si content should be controlled within an optimal range to balance its strengthening effect and embrittlement tendency.

**Figure 6:**
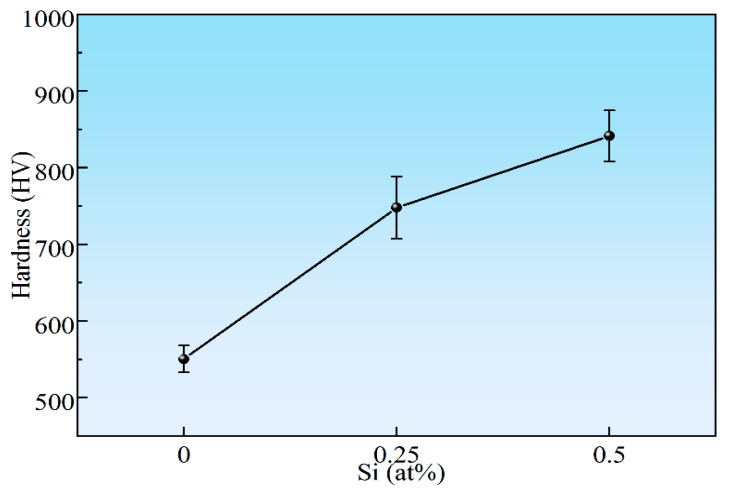
Microhardness of the NbMoTaWSi*_x_* RHEAs.

Figure 6 shows the Vickers microhardness values of the NbMoTaWSi*_x_* RHEAs. For the NbMoTaW RHEA, the hardness is 550.65 HV. The hardness of the NbMoTaWSi_0.25_ and NbMoTaWSi_0.5_ alloys is about 747.94 HV and 841.53 HV, respectively, which can be demonstrated by the hardness of the NbMoTaWSi*_x_* RHEAs increasing with the increase of Si volume fraction. This result is consistent with the findings of Guo regarding the formation of intermetallic silicide in the alloy system [45]. The study suggests that microalloying with Si represents a viable strategy for significantly enhancing the strength and hardness of RHEAs at both room temperature (RT) and elevated temperatures. This improvement stems from silicide formation, which strengthens the matrix and consequently increases alloy hardness.

**Figure 7:**
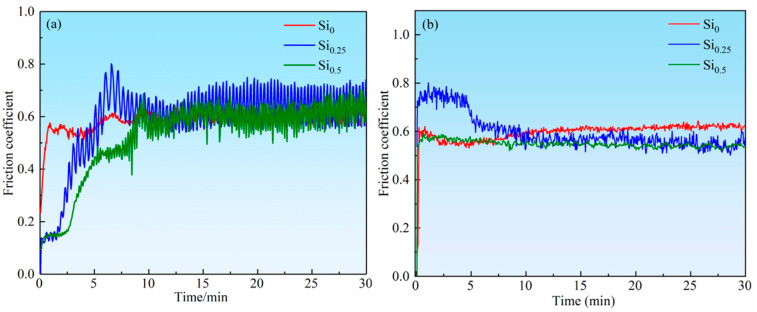
The change curve of friction coefficients of NbMoTaWSi*_x_* RHEAs during wear from different counterbody materials: (**a**) GCr15 ball, (**b**) Si_3_N_4_ ball.

Figure 7 shows the friction coefficient curves of NbMoTaWSi*_x_* alloys against different counterbody materials. The wear tests employed a load of 10 N, a speed of 500 r/min, a wear radius of 2 mm, and a duration of 30 min. Under the above experimental conditions, when the counterbody material is a GCr15 steel ball, the friction coefficients of the alloy fluctuate significantly around a value of 0.6, as seen in Figure 7a. When the counterbody material is Si_3_N_4_, the friction coefficient curve of the alloy is relatively smooth (as shown in Figure 7b). Silicide hard phases fracture into abrasive particles during friction. The continuous generation and expulsion of these particles at the interface causes periodic friction coefficient variations. Simultaneously, differing wear rates between the matrix and silicides induce dynamic changes in surface roughness. Moreover, local flash temperatures (potentially reaching several hundred degrees Celsius) may occur during the friction process, which trigger the formation and spallation of oxide films, constituting another source of fluctuation. Such fluctuations are normal and instead demonstrate the reliability of the experimental data. Comparative results indicate that the silicon content has little effect on the friction coefficient of the NbMoTaW_6_ RHEA at room temperature.

**Figure 8:**
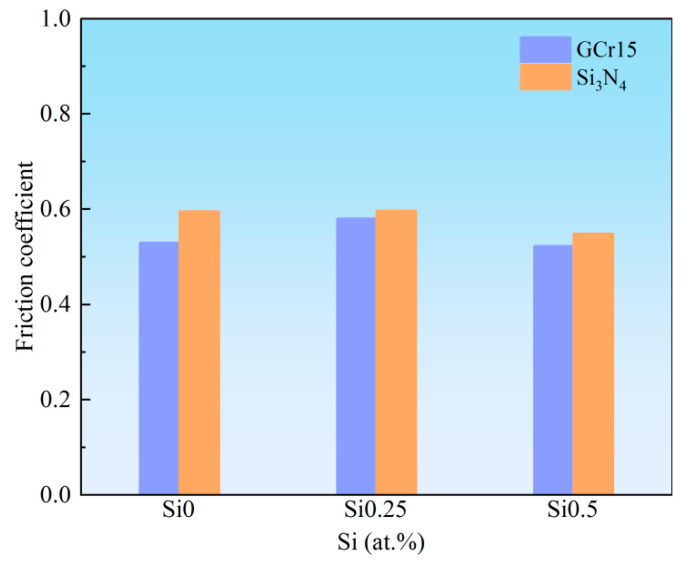
Comparative diagrams of average friction coefficients of NbMoTaWSi*_x_* RHEAs under different counterbody materials.

Comparative diagrams of average friction coefficients of NbMoTaWSi*_x_* RHEAs under different counterbody materials are given in Figure 8. It can be observed that there is no obvious change in the average friction coefficient of alloys with different Si content under the same counterbody materials. The average friction coefficient obtained with GCr15 steel balls is greater than that obtained with Si_3_N_4_ ceramic balls. This difference between the two counterbody materials should be attributed to the higher hardness of Si_3_N_4_, which can resist wear from NbMoTaW_6_ RHEAs during friction. 

**Figure 9:**
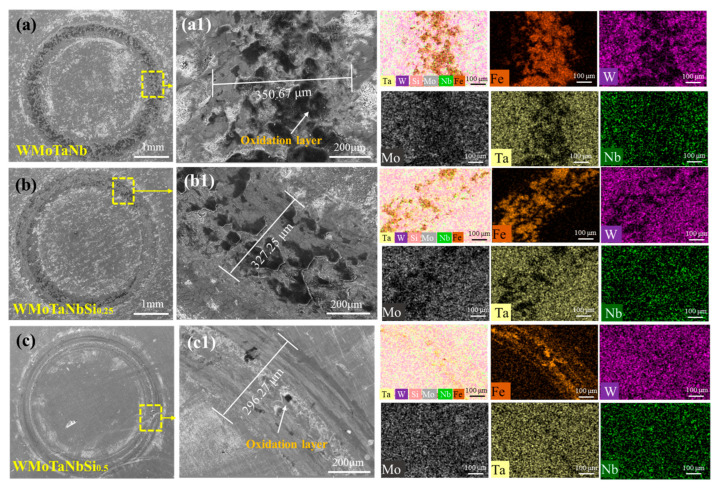
SEM micrographs and EDS mapping of worn surfaces of NbMoTaWSi*_x_* alloys under GCr15 counter ball material: (**a**,**a1**) *x* = 0, (**b**,**b1**) *x* = 0.25, and (**c**,**c1**) *x* = 0.5.

In order to investigate the wear behavior of NbMoTaWSi*_x_* RHEAs with different counter ball materials, the wear abrasion morphology, wear tracks, and chemical composition of different areas of the abrasion surface were analyzed. Figure 9 shows the SEM micrographs and EDS mapping of worn surfaces of NbMoTaWSix RHEAs with GCr15 counter ball material. Figure 9a–c show the low magnification images of the wear tracks about the NbMoTaWSi*_x_* RHEAs with different Si content. It can be seen that the wear widths of NbMoTaW, NbMoTaWSi_0.25_, and NbMoTaWSi_0.5_ RHEAs are 350.67 μm, 327.25 μm, and 296.27 μm, respectively (see Figure 9(a1–c1)). The narrow width of the wear trace can be attributed to the increase in the hardness of RHEAs with the increased Si content. At the same time, the wear resistance of the alloy increases. Moreover, according to the microscopic morphology of the wear scars, adhesive wear features are revealed in the Si-free alloy, while an oxide transfer layer is formed on their surfaces. With the increase of Si content, the adhesive wear of the alloy decreases, and the black oxidized glaze layer at the wear marks decreases. When the content of Si increases to *x* = 0.5, the wear marks of the alloy are obviously smooth. Ploughing effects at the contact interface between the counterbody and alloy surface generate grooves aligned with the sliding direction and cause material-induced darkening on the contact surface of the counterbody. Combined with EDS analysis, with increasing Si addition, the material transfer of Fe on the wear track becomes shallower. This phenomenon arises because silicides enhance the strength and hardness of RHEAs [46], thereby suppressing the adhesive wear of RHEAs and consequently improving the wear resistance of the alloy.

**Figure 10:**
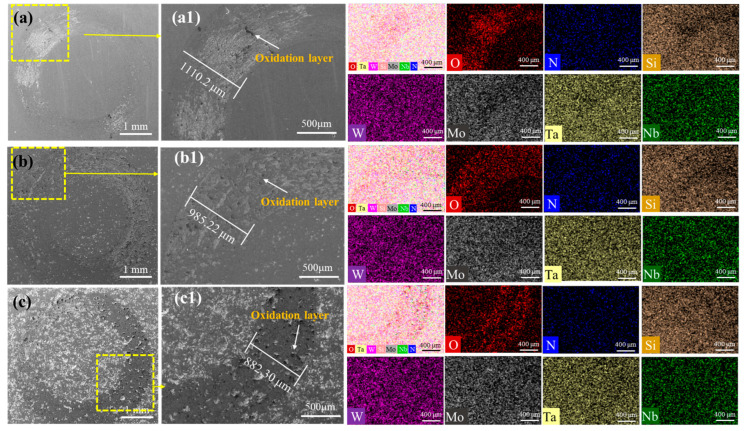
SEM micrographs and EDS mapping of worn surfaces of NbMoTaWSix alloys under Si_3_N_4_ counter ball material: (**a**,**a1**) *x* = 0, (**b**,**b1**) *x* = 0.25, and (**c**,**c1**) *x* = 0.5.

Figure 10 shows the SEM micrographs and EDS mapping of the worn surfaces of NbMoTaWSix RHEAs with Si_3_N_4_ counter ball material; the wear track widths of RHEAs with varying Si contents measured 1110.02 μm, 985.22 μm, and 882.30 μm, respectively. A significant reduction in wear track width was observed with increasing Si content. EDS analysis revealed shallower wear tracks and smoother surfaces at higher Si concentrations (Figure 10(a1–c1)). When counterface hardness substantially exceeds that of the alloy, wear rates become primarily dependent on the material’s intrinsic hardness rather than counterface hardness. Consistent with Wayne and Buckley’s model [47], the enhanced wear resistance of Si-containing RHEAs correlates directly with increased hardness. The formation of the oxide layer on the surface of the wear tracks can also be observed, indicating that the oxidation reaction occurred during the wear process. The reduction of the black oxidized glaze layer on the wear surface and the relative smoothness of the alloy surface can be directly observed under different counter ball materials, indicating that Si addition can effectively alleviate the oxidation.

The preceding discussion indicates that the primary wear mechanisms of RHEAs involve adhesive wear and oxidative wear. Table 3 presents the EDS analysis of distinct regions on the wear track surfaces of NbMoTaWSi*_x_* RHEAs. Notably, the oxygen content within the wear tracks exceeds that of the surrounding regions, with areas of elevated oxygen concentrations exhibiting reduced proportions of metallic elements. This observation suggests localized oxide formation through tribo-oxidation reactions, which depletes metallic constituents. It can also be found that the content of O at the wear track decreases with Si addition. These results suggested that the Si addition can effectively alleviate the oxidation.

**Figure 11:**
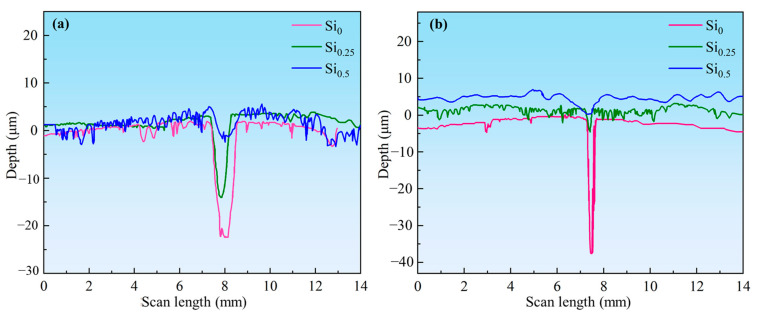
Comparison of worn depths of NbMoTaWSi*_x_* RHEAs after wear from different counter ball materials: (**a**) GCr15 ball, (**b**) Si_3_N_4_ ball.

The wear track depths of NbMoTaWSix RHEAs after testing with different counterface materials are shown in Figure 11. The worn depth of the NbMoTaW RHEA was approximately 25 μm, which decreased significantly upon Si addition (seen in Figure 11a). Comparative analysis of wear depths across counterface materials reveals that Si-containing alloys exhibit superior wear resistance relative to the matrix alloy. This enhancement arises from a Si-induced hardness improvement, which directly enhances the material’s wear resistance. Furthermore, wear tracks generated by Si_3_N_4_ counterparts demonstrate notably narrower profiles compared to those produced by GCr15 steel balls (Figure 11b). This difference stems from the significantly higher hardness of Si_3_N_4_ (HV 1600) versus the GCr15 steel ball, which reduces abrasive penetration during wear testing. The observed results confirm that Si_3_N_4_ counterfaces provide more reliable interfacial conditions for investigating RHEA wear mechanisms.

## 4. Conclusions

In this investigation, NbMoTaWSi*_x_* RHEAs were synthesized via vacuum induction levitation melting (VILM). A systematic characterization of their microstructural evolution and mechanical performance yielded the following key findings:

(1) The NbMoTaW RHEA exhibits a single BCC structure. Si addition induces the formation of the (Nb,Ta)_5_Si_3_ phase, and the volume fraction of the silicide phase increases with higher Si content.
(2) Si addition significantly improves the strength and hardness of the NbMoTaWSi*x* RHEA. However, the plasticity deteriorates with increasing silicide phase content. The fracture mechanism of Si-containing RHEAs reveals brittle fracture behavior, primarily governed by the synergistic interaction between the silicide and matrix fracture modes.
(3) The wear mechanism of NbMoTaWSi*_x_* RHEAs involves adhesive wear and oxidative wear. Wear resistance is enhanced by Si addition, attributable to an improved hardness and oxidation resistance. Tribological evaluation under different counterface materials demonstrates that Si_3_N_4_ counterfaces are more suitable for studying RHEA wear mechanisms.

## Figures and Tables

**Table 1:** The radius of different elements in RHEAs [41].

Element	W	Mo	Ta	Nb	Si
Radius (Å)	1.41	1.40	1.48	1.48	1.11

**Table 2:** Compressive mechanical properties of NbMoTaWSi*_x_* RHEAs.

*σ*_p_ (MPa)	*E* (GPa)	*σ*_0.2_ (MPa)	Alloy
1029.35	20.07	1029.36	WMoTaNb
2519.69	26.53	2560.45	WMoTaNbSi_0.25_
1613.36	24.94	1560.83	WMoTaNbSi_0.5_

**Table 3:** Chemical composition of the NbMoTaWSi*_x_* RHEAs constituent (at.%).

N	Si	Nb	Ta	Mo	W	O	Region	Alloy
3.0	6.3	5.6	6.6	7.1	2.0	69.4	Inside	WMoTaNb
1.6	9.2	17.2	18.6	19.2	23	11.2	Outside	
3.2	9.2	6.3	7.6	7.4	5.7	60.6	Inside	WMoTaNbSi_0.25_
3.4	6.0	14	24.4	27.5	12.0	12.7	Outside	
3.7	14.1	7.1	8.0	8.0	5.6	53.5	Inside	WMoTaNbSi_0.5_
3.3	9.6	18.8	19.2	27.3	12.2	9.6	Outside	

## Data Availability

The original contributions presented in this study are included in the article. Further inquiries can be directed to the corresponding authors.
